# A new “surgical emergency care bundle power plan” improves physician prescribing compliance for emergency surgical admissions

**DOI:** 10.1186/s13037-026-00498-8

**Published:** 2026-07-25

**Authors:** Ahmed Tawfik, Olivia Szepietowski, Faria Antara, Aisha Ehsan

**Affiliations:** Department of General Surgery, Ashford and St Peter’s Hospital NHS Foundation Trust, Guildford Road, Chertsey, KT16 0PZ UK

## Abstract

**Background:**

Medication errors and omissions remain a significant source of preventable harm in emergency surgical care. Structured electronic order sets (“power plans”) may improve compliance with evidence-based prescribing practices. This study evaluated the impact of a new “surgical emergency care bundle power plan” on prescribing compliance in emergency surgical admissions.

**Methods:**

A prospective two-cycle quality improvement audit was conducted across two hospital sites within a UK NHS Foundation Trust. All emergency surgical admissions during a defined one-month audit period were included. Compliance was defined as prescribing essential medications and requesting investigations within six hours of the documented decision to admit. Following baseline data collection, a structured electronic order set (“surgical emergency care bundle power plan”) was implemented within the Cerner Millennium electronic prescribing system, supported by education and training. A second audit cycle was subsequently performed using identical methodology.

**Results:**

A total of 560 emergency surgical admissions were included across the two audit cycles (Cycle 1: *n* = 280; Cycle 2: *n* = 280). Baseline compliance was low across several prescribing domains. Following implementation of the power plan, compliance improved across all measured parameters: VTE prophylaxis (25% to 70%), analgesia (60% to 82%), antiemetics (28% to 61%), gastric protection (44% to 46%), antibiotics (68% to 82%), intravenous fluids (72% to 90%), laxatives (8% to 60%), nutritional supplementation (5% to 70%), and next-day blood test requests (5% to 60%).

**Conclusions:**

Implementation of the “surgical emergency care bundle power plan” was associated with clinically meaningful improvements in prescribing compliance across multiple domains of emergency surgical care. Structured electronic order sets represent an effective, scalable quality improvement intervention capable of reducing omission errors and supporting evidence-based clinical practice.

**Supplementary Information:**

The online version contains supplementary material available at https://doi.org/10.1186/s13037-026-00498-8.

## Background

Medication errors remain among the most common causes of preventable harm in hospitalised patients [1, 2] and are particularly relevant in emergency surgical admissions, where clinicians must make multiple time-critical decisions under significant workload pressures. Emergency surgical patients frequently require the prompt initiation of analgesia, intravenous fluids, venous thromboembolism (VTE) prophylaxis, antibiotics, antiemetics, nutritional support, and appropriate investigations. Failure to prescribe these interventions in a timely manner may adversely affect patient outcomes and contribute to avoidable complications.

The widespread adoption of electronic prescribing systems has created opportunities to improve medication safety through standardisation and clinical decision support. Computerised provider order entry (CPOE) systems and structured order sets have been associated with reductions in prescribing errors, improved adherence to evidence-based guidelines, and enhanced workflow efficiency [3–6]. Several studies have demonstrated improvements in VTE prophylaxis compliance, antimicrobial stewardship, and medication safety following implementation of electronic prescribing interventions [7–9]. 

Despite these advances, prescribing omissions remain common in acute surgical care [10]. The emergency admission process is inherently complex, involving unselected patients with a broad range of diagnoses and varying clinical acuity. Consequently, clinicians remain vulnerable to omissions, particularly during busy on-call periods. Structured admission order sets may provide a practical solution by prompting consideration of commonly required medications and investigations while preserving clinician autonomy and individualised decision-making.

The objective of this quality improvement project was to assess the timeliness and completeness of prescribing essential medications and investigations in emergency surgical admissions and to evaluate the impact of implementing a structured electronic admission order set, the “surgical emergency care bundle power plan”.

## Methods

### Study design and setting

A prospective two-cycle quality improvement audit was conducted at Ashford and St Peter’s Hospitals NHS Foundation Trust across both Ashford Hospital and St Peter’s Hospital sites.

The baseline audit cycle was conducted between January 2026 and February 2026. Following implementation of the “surgical emergency care bundle power plan” and supporting educational interventions, a second audit cycle was performed between March 2026 and April 2026 using identical methodology.

### Study population

All patients admitted under the on-call emergency surgical team during the defined audit periods were included. Patients were admitted via the Emergency Department, Same Day Emergency Care pathway, or direct emergency referral routes. No exclusion criteria were applied.

### Study hypothesis

We hypothesised that implementation of a structured electronic admission order set (“surgical emergency care bundle power plan”) would improve compliance with timely prescribing of essential medications and investigations in emergency surgical admissions.

### Data collection

Data were collected prospectively from the Cerner Millennium electronic patient record system. The documented decision-to-admit (D2A) time served as the reference point for assessment of prescribing compliance.

Medication prescriptions and investigation requests were reviewed and compared against the D2A timestamp. Compliance was defined as completion within six hours of admission.

### Intervention

The “surgical emergency care bundle power plan” was developed within the Cerner Millennium electronic prescribing system to facilitate structured prescribing at the point of admission.

The power plan included prompts for:

- Venous thromboembolism prophylaxis
- Intravenous fluids
- Analgesia
- Antiemetics
- Antibiotics (where clinically indicated)
- Gastric protection
- Nutritional supplementation
- Laxatives
- Next-day blood investigations

The intervention was supported by structured induction sessions, educational meetings, and dissemination of guidance materials.

Importantly, the power plan was designed to support rather than replace clinical decision-making. Clinicians retained full discretion to modify, omit, or add medications according to individual patient circumstances.

### Outcome measures

The primary outcome was compliance with prescribing essential medications within six hours of the documented decision to admit.

VTE prophylaxis compliance was assessed only where pharmacological thromboprophylaxis was clinically indicated. Patients with documented contraindications, active bleeding, therapeutic anticoagulation, imminent surgery, or other appropriate clinical reasons for withholding prophylaxis were considered appropriately managed and were not classified as non-compliant.

Antibiotic prescribing was assessed only when antibiotic therapy was clinically indicated.

Gastric protection prescribing was evaluated where clinically appropriate according to local prescribing guidance. The power plan did not mandate proton pump inhibitor prescription but provided prompts for clinician consideration where appropriate.

### Statistical analysis

Compliance rates were reported as percentages before and after implementation of the intervention. Given the quality improvement nature of the project and the focus on process compliance rather than patient-level clinical outcomes, descriptive statistical methods were used to evaluate changes in prescribing performance between audit cycles. Formal hypothesis testing was not performed.

### Ethics

This project was conducted as a quality improvement audit/service evaluation and did not require formal Research Ethics Committee approval under NHS Health Research Authority guidance.

## Results

### Baseline findings

A total of 560 emergency surgical admissions were included across the two audit cycles. The baseline audit cycle (Cycle 1) included 280 emergency surgical admissions, while the post-intervention audit cycle (Cycle 2) included 280 emergency surgical admissions.

Baseline compliance demonstrated significant variation across prescribing domains.

The lowest compliance was observed for nutritional supplementation (5%), next-day blood test requests (5%), laxatives (8%), and VTE prophylaxis (25% of eligible patients). Baseline rates for all nine prescribing domains are summarised in Table 1.

These findings identified multiple opportunities for improvement and suggested that prescribing omissions represented a system-wide rather than individual clinician issue.

### Post-intervention findings

Following implementation of the “surgical emergency care bundle power plan”, improvements were observed across all measured parameters.

**Table 1:** Prescribing compliance before and after implementation of the surgical emergency care bundle power plan, ordered by absolute improvement

Medication / Parameter	Baseline	Post-intervention	Absolute improvement
Nutritional supplementation	5%	70%	+ 65%
Next-day blood requests	5%	60%	+ 55%
Laxatives	8%	60%	+ 52%
VTE prophylaxis	25%	70%	+ 45%
Antiemetics	28%	61%	+ 33%
Analgesia	60%	82%	+ 22%
Intravenous fluids	72%	90%	+ 18%
Antibiotics	68%	82%	+ 14%
Gastric protection	44%	46%	+ 2%

Compliance defined as prescribing within six hours of the documented decision to admit. VTE prophylaxis, antibiotic, and gastric protection compliance assessed only where clinically indicated

**Fig. 1:**
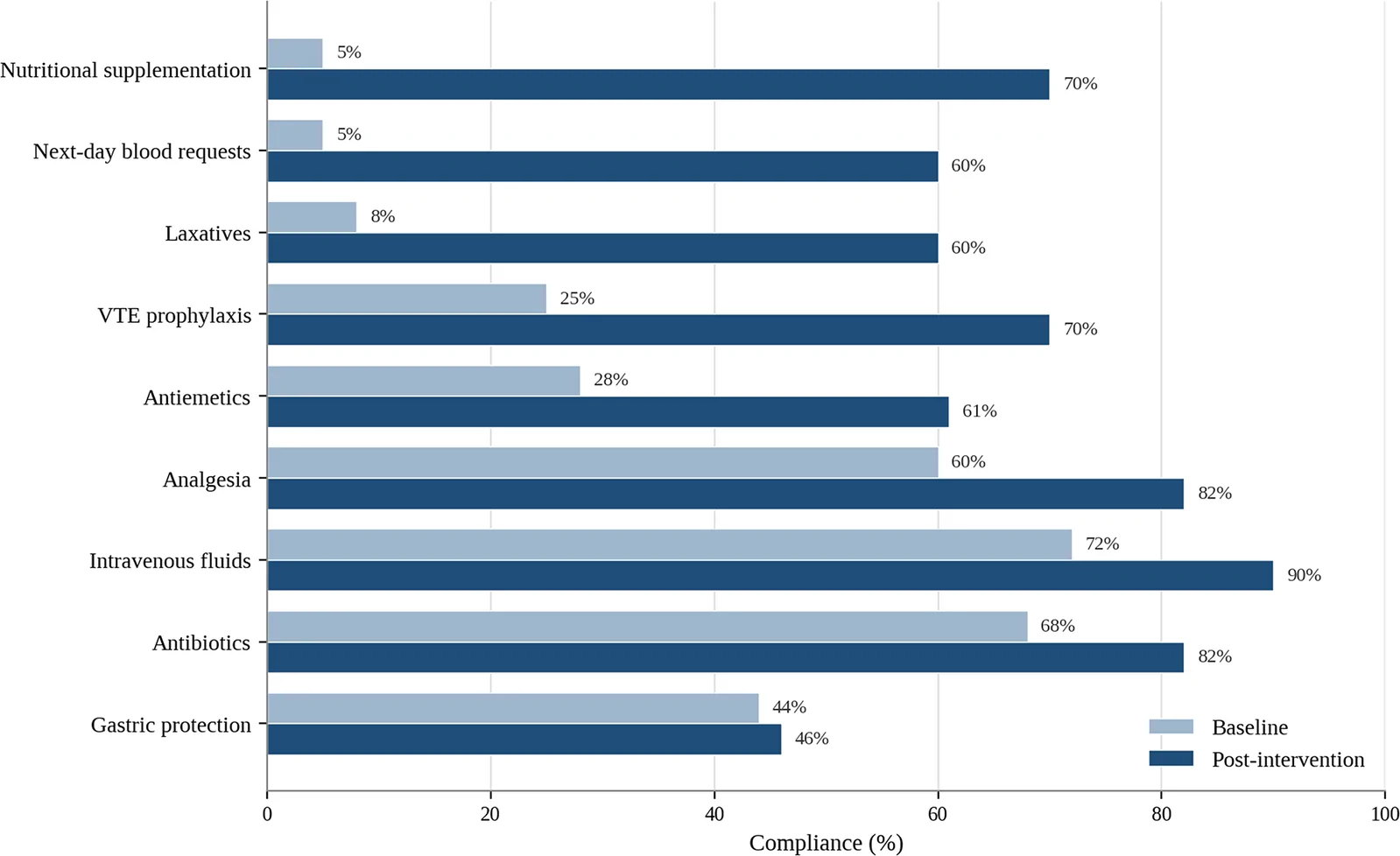
Prescribing compliance before and after power plan implementation, ordered by absolute improvement

A graphical representation of prescribing compliance before and after implementation of the power plan is shown in Fig. 1.

The largest improvements were observed in nutritional supplementation, blood test requests, laxative prescribing, and VTE prophylaxis. Gastric protection demonstrated only modest improvement, reflecting more selective prescribing practices and clinical discretion.

Overall, the intervention was associated with substantial improvements in prescribing compliance across emergency surgical admissions.

## Discussion

### Principal findings

This quality improvement project demonstrated substantial improvements in prescribing compliance following implementation of a structured electronic admission order set. Significant gains were observed across multiple prescribing domains, particularly VTE prophylaxis, nutritional supplementation, blood test requests, and laxative prescribing.

These findings suggest that many prescribing omissions are related to system and workflow factors rather than deficiencies in clinical knowledge. By providing structured prompts at the point of admission, the power plan reduced the likelihood that important medications and investigations would be overlooked during busy emergency surgical admissions.

### Comparison with existing literature

Our findings are consistent with previous studies demonstrating the benefits of computerised provider order entry systems and clinical decision support tools [4, 5, 11–13]. Electronic order sets have been associated with improved compliance with evidence-based guidelines, reductions in prescribing errors, and enhanced medication safety [6, 14, 15]. 

Several studies have shown improvements in VTE risk assessment and thromboprophylaxis prescribing following implementation of electronic reminders and bundled interventions [7, 16, 17]. Similar benefits have been reported for antimicrobial stewardship initiatives and standardised admission pathways [8]. 

The present study extends these findings to emergency surgical admissions, a clinical environment characterised by high workload, diagnostic uncertainty, and time-sensitive decision-making.

### Clinical implications

A major strength of this intervention is its simplicity and scalability. The power plan was implemented using existing Cerner Millennium functionality and therefore required minimal additional financial investment.

Although termed a “surgical emergency care bundle power plan”, the intervention was not intended to replace disease-specific pathways. Rather, it functioned as a generic admission bundle designed to ensure consideration of commonly required medications and investigations while preserving clinician autonomy and individualised patient management.

Importantly, the power plan was designed to support rather than replace clinical judgement. Prescribers retained full discretion regarding medication selection and could modify, omit, or add prescriptions according to individual patient needs.

The power plan incorporated decision-support prompts for venous thromboembolism prophylaxis, including contraindications to pharmacological prophylaxis and weight- and renal function-adjusted dosing recommendations. Similarly, gastric protection prompts were designed to support clinician assessment rather than mandate prescribing, ensuring that individual patient risk factors remained central to clinical decision-making.

Although gastric protection prompts were incorporated within the power plan, these were intended only to prompt clinician consideration rather than mandate prescription. Future studies should evaluate whether admission power plans influence discharge prescribing practices and assess the appropriateness of long-term proton pump inhibitor continuation following hospitalisation.

### Limitations

This study has several limitations. First, it was conducted within a single NHS Foundation Trust, potentially limiting generalisability. Second, the before-and-after audit design introduces the possibility of confounding and temporal bias. Third, prescribing compliance was assessed as a process measure rather than patient-centred outcomes such as hospital-acquired VTE, infection rates, length of stay, or mortality. Fourth, the sustainability of improvements beyond the second audit cycle remains unknown. Finally, the study did not evaluate discharge prescribing practices, particularly continuation of proton pump inhibitors after discharge, which represents an important area for future investigation.

## Conclusions

Implementation of the “surgical emergency care bundle power plan” was associated with clinically meaningful improvements in prescribing compliance across multiple domains of emergency surgical care. Structured electronic order sets appear to be an effective, scalable, and reproducible quality improvement intervention capable of reducing omission errors and supporting evidence-based clinical practice.

Further evaluation should assess long-term sustainability and determine whether improvements in prescribing compliance translate into measurable benefits in patient outcomes.

## Supplementary Information

Below is the link to the electronic supplementary material.

Supplementary Material 1

Supplementary Material 2

## Data Availability

The data analysed during this quality improvement project are not publicly available due to institutional governance and patient confidentiality considerations.
