# Supplementary material for: A new “surgical emergency care bundle power plan” improves physician prescribing compliance for emergency surgical admissions
Source: Patient Saf Surg. 2026 Jul 25;20:42. doi: 10.1186/s13037-026-00498-8 (PMC13741569; doi:10.1186/s13037-026-00498-8)
Supplement: Supplementary file 1 [file 13037_2026_498_MOESM1_ESM.docx]

**Supplementary Appendix 1**

Full-resolution screenshots of the surgical emergency care bundle powerplan as configured within the Cerner Millennium electronic prescribing system, shown in the order presented to the prescriber. Identifiable patient information has been removed.

**Figure S1.1.** Powerplan overview showing the medications phase, with the embedded venous thromboembolism (VTE) prophylaxis care plan and the non-opioid analgesia options (paracetamol, with a prompt to consider gastric protection, and ibuprofen).

**Figure S1.2.** Opioid analgesia, including regular dihydrocodeine, breakthrough options (first-line morphine, second-line oxycodone), a naloxone order prompted for all patients as opioid-toxicity cover, and patient-controlled analgesia (PCA).

**Figure S1.3.** Antiemetic options (ondansetron, cyclizine, metoclopramide) and gastric protection options (pantoprazole, lansoprazole, famotidine, omeprazole).

**Figure S1.4.** Antimicrobial section with an embedded link to the Trust antimicrobial guidelines, covering agents for intra-abdominal and hepatobiliary indications and alternatives for patients with penicillin allergy (temocillin, teicoplanin).

**Figure S1.5.** Intravenous fluid options (sodium chloride 0.9%, Plasma-Lyte 148, potassium chloride-containing solutions) and laxatives (lactulose, macrogol, senna, docusate, sodium phosphate enema).

**Figure S1.6.** Nutritional supplementation (oral supplement drinks and vitamins) and the start of the laboratory monitoring section, including therapeutic drug-level monitoring and baseline blood tests scheduled at six hours.

**Figure S1.7.** Laboratory investigations requested at six hours after admission, including full blood count, C-reactive protein, liver function tests, amylase, urea and electrolytes, coagulation screen, group and antibody screen, and bone profile.
