# Supplementary material for: A new “surgical emergency care bundle power plan” improves physician prescribing compliance for emergency surgical admissions
Source: Patient Saf Surg. 2026 Jul 25;20:42. doi: 10.1186/s13037-026-00498-8 (PMC13741569; doi:10.1186/s13037-026-00498-8)
Supplement: Supplementary file 2 [file 13037_2026_498_MOESM2_ESM.docx]

**Supplementary Appendix 2**

Screenshots of the venous thromboembolism (VTE) prophylaxis care plan embedded within the powerplan, showing the decision-support prompts and the weight- and renal-function-stratified low-molecular-weight heparin dosing. Identifiable patient information has been removed.

**Figure S2.1.** Thromboprophylaxis decision support: the timing prompt for patients admitted after 18:00 (highlighted) and the list of contraindications to pharmacological VTE prophylaxis.

**Figure S2.2.** Weight-banded enoxaparin dosing for patients with creatinine clearance greater than or equal to 30 mL/min, stratified by body-weight category.

**Figure S2.3.** Enoxaparin dosing for higher body-weight categories and for reduced renal function (creatinine clearance 15-30 and below 15 mL/min), including the unfractionated heparin option.
